# Supplementary material for: Vaccination with Schistosoma mansoni Cholinesterases Reduces the Parasite Burden and Egg Viability in a Mouse Model of Schistosomiasis
Source: Vaccines (Basel). 2020 Apr 3;8(2):162. doi: 10.3390/vaccines8020162 (PMC7349746; doi:10.3390/vaccines8020162)
Supplement: Supplementary file 1 [file vaccines-08-00162-s001.pdf]

Supporting information

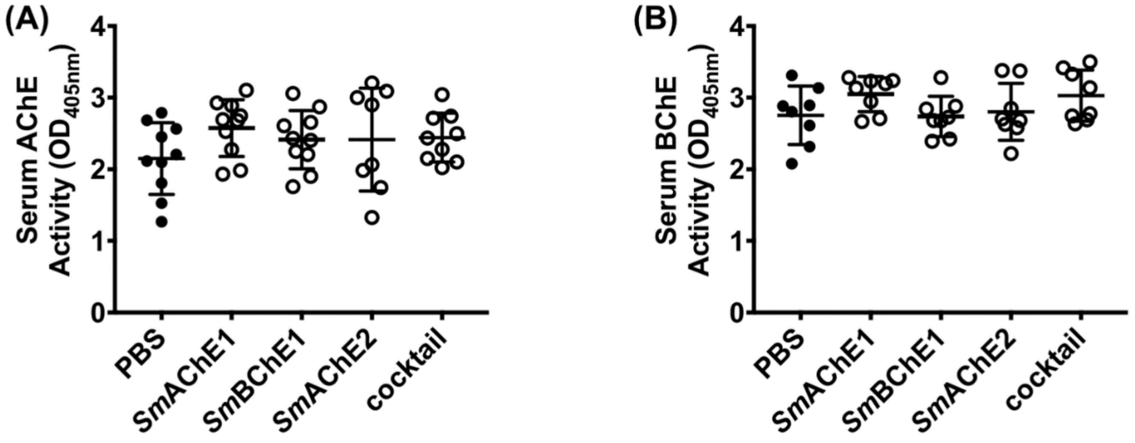

Figure S1. ChE activity in the serum of control and vaccinated mice (trial 2). Activity was measured by Ellman assay. (A) AChE. (B) BChE. Data are the average of triplicate technical experiments ± SEM.

Table S1. Anti-*Sm*ChE IgG titers and worm burdens of vaccinated groups from trial 1.

**Immunogen**	**Mouse**	**Total IgG endpoint titres**		**Worm burden**
		**Pre-challenge**	**Necropsy**	
*Sm*AChE1 n = 10	1	1,974,491	229,723	29
	2	682,718	134,438	39
	3	866,872	186,065	30
	4	1,260,333	163,544	36
	5	2,129,001	171,513	34
	6	1,173,196	299,021	21
	7	2,096,392	113,649	17
	8	1,597,200	188,144	40
	9	1,692,421	210,666	19
	10	1,638,522	153,495	35
*Sm*BChE1 n = 10	1	516,530	106,352	30
	2	1,430,114	105,517	42
	3	540,784	120,551	19
	4	985,449	79,625	18
	5	1,617,861	75,449	29
	6	955,805	74,614	28
	7	1,609,776	28,676	39
	8	1,223,502	83,244	21
	9	1,275,604	59,579	20
	10	1,257,638	117,210	21
*Sm*AChE2 n = 9	1	485,262	120,273	37
	2	488,682	178,182	40
	3	495,869	77,676	34
	4	510,242	94,102	37
	5	495,869	143,659	30
	7	492,275	49,835	20
	8	560,547	197,392	23
	9	538,988	163,426	15
	10	510,242	96,886	20
*Sm*ChE cocktail n=10	1	919,872	154,517	40
	2	1,616,963	173,170	29
	3	1,185,773	165,096	28
	4	2,378,732	309,590	34

	5	1,381,605	172,335	37
	6	1,622,353	448,795	17
	7	1,192,959	97,165	21
	8	1,500,182	199,341	23
	9	3,171,044	449,630	19
	10	2,112,832	351,073	21

Table S2. Anti-*Sm*ChE IgG titers and worm burdens of vaccinated groups from trial 2.

**Immunogen**	**Mouse**	**Total IgG endpoint titres**		**Worm burden**
		**Pre-challenge**	**Necropsy**	
*Sm*AChE1 n = 10	1	547,971	168,394	32
	2	2,355,376	86,969	21
	3	923,466	124,390	20
	4	1,131,874	110,877	18
	5	2,966,229	128,548	30
	6	1,166,010	199,579	20
	7	1,947,542	135,824	21
	8	2,089,475	118,153	22
	9	1,764,286	145,180	19
	10	2,711,108	114,342	20
*Sm*BChE1 n = 10	1	284,276	71,273	9
	2	449,954	141,988	20
	3	396,539	176,790	19
	4	522,381	124,170	28
	5	536,866	102,176	19
	6	1,879,484	89,648	18
	7	451,764	36,750	22
	8	899,002	108,579	30
	9	716,123	125,005	18
	10	588,470	156,466	20
*Sm*AChE2 n = 8	1	1,130,077	430,420	17
	2	2,076,899	190,432	10
	3	1,404,961	338,545	32
	5	1,609,776	319,056	26
	7	1,814,592	249,733	19
	8	2,427,241	270,056	25
	9	1,805,609	288,153	16
	10	2,136,188	248,062	15
*Sm*ChE cocktail n = 9	1	2,567,378	364,715	28
	2	1,951,135	387,173	20
	3	1,352,859	361,122	15
	4	1,891,847	720,447	14
	5	1,245,061	363,817	16
	6	2,005,034	1,370,825	21
	7	1,478,623	293,748	19
	9	3,066,840	1,105,823	11
	10	1,999,644	893,821	24
